# *Loss of the RAD-51 isoform A* redirects DNA repair and preserves genome stability in FANCD2-deficient *Caenorhabditis elegans*

**DOI:** 10.1186/s12915-026-02652-1

**Published:** 2026-06-23

**Authors:** Alessandra Masullo, Marcello Germoglio, Rosanna Mattossovich, Claudia Esposito, Anna Valenti, Adele Adamo

**Affiliations:** 1 Institute of Bioscience and BioResources, National Research Council of Italy (CNR), Via Pietro Castellino 111, Naples, 80131 Italy; 2 Institute of Biochemistry and Cell Biology, National Research Council of Italy (CNR), Via Pietro Castellino 111, Naples, 80131 Italy

**Keywords:** Genome instability, Fanconi anemia, Homologous recombination, Meiosis, Model organism

## Abstract

**Background:**

Fanconi anemia is a rare genomic instability syndrome associated with congenital abnormalities and cancer predisposition. These alterations are mainly due to deficiencies in DNA repair mechanisms. The Fanconi anemia pathway is evolutionarily conserved, allowing functional studies in model organisms like *Caenorhabditis elegans*, where it promotes and executes error-free homologous-recombination over the mutagenic non-homologous-end-joining pathway; *fcd-2* (FANCD2 ortholog) plays a key role in this regulation to preserve genome stability. In this study, we report that the choice of DNA repair pathway for resolving double-strand breaks is influenced by absence of the RAD-51 long isoform, a key component of the Fanconi anemia pathway that plays a central role in homologous strand exchange during recombination.

**Results:**

In *C. elegans*, which is predicted to encode three RAD-51 isoforms, we find that loss of RAD-51 isoform A enhances homologous recombination efficiency. Additionally, RAD-51 isoform A depletion decreases developmental and meiotic defects in *fcd-2* mutants as well as reduces chromosome aggregation in *fcd-2*-deficient germ cells.

**Conclusions:**

Together, these findings reveal a novel role for RAD-51 *C. elegans*, suggesting that the pattern of RAD-51 isoform expression modulates the balance between homologous recombination and non-homologous-end-joining, thereby preserving genome stability.

**Supplementary Information:**

The online version contains supplementary material available at https://doi.org/10.1186/s12915-026-02652-1.

## Background

Fanconi anemia (FA) is a rare genomic instability disease caused by mutations in at least 23 genes. It is characterized by congenital abnormalities, bone marrow failure, endocrine dysfunction, and cancer predisposition [1–4]. The clinical presentation of Fanconi anemia is highly heterogeneous, with manifestations that can involve nearly all organ systems [5]. Hematological features include aplastic anemia and a significantly increased risk of leukemia and myelodysplastic syndromes. Additionally, patients exhibit a heightened susceptibility to squamous cell carcinomas, particularly of the head and neck, as well as gynecological malignancies in females [5, 6].

FA proteins are involved in the repair of different types of DNA damage. Specifically, it has been linked to inter-strand cross-link (ICL) repair [7], which is generated by alkylating agents that interfere with DNA replication and transcription [8]. ICL lesions, which affect both strands of the DNA, require efficient and accurate repair mechanisms that are differentially activated depending on the specific phase of the cell cycle [4]. During the S phase, ICLs are recognized by the “Anchor complex” containing FANCM protein, which in turn mediates the assembly of the “core complex” composed of multiple subunits, including seven FA proteins (FANCA, B, C, E, F, G, L). The role of this complex is to mono-ubiquitinate FANCD2 and FANCI, facilitating their formation into the ID2 heterodimer (ID2 complex) and to promote the ICL unhooking through the action of two additional FA proteins, FANCDQ/XPF and FANCP/SLX4. These events lead to a nick in the DNA strand flanking the ICL and produce an ICL-derived double strand break (DSB) [9–11].

The repair of DSBs is a critical step in preventing genome instability and for cell survival. DSBs are repaired by two main mechanisms: non-homologous end joining (NHEJ) or homologous recombination (HR). NHEJ is a potentially error-prone mechanism that generates base loss and/or chromosomal rearrangements: the heterodimer KU70/80 recognizes the DSB ends, protects them from degradation, and recruits the DNA ligase LIG IV [12], which joins the ends independently of their homology [13].

HR is an error-free repair mechanism that requires a DNA homologous template and is mainly active during the meiotic DSB repair, when it is crucial to prevent genome instability. In meiosis, HR is also important to ensure crossover (CO) formation, which is essential for the accurate segregation of homologous chromosomes [14].

During meiotic prophase I, HR is a tightly regulated process essential for the accurate segregation of homologous chromosomes and the maintenance of genome stability. It is initiated in early prophase I by the programmed induction of DSBs catalyzed by the evolutionarily conserved topoisomerase-like enzyme SPO11 [15]. Following DSB formation, the DNA ends undergo resection to produce 3′ single-stranded DNA (ssDNA) overhangs, which are rapidly coated by replication protein A (RPA) [16]. Subsequently, RPA is replaced by the recombinase RAD51 [14], which facilitates the invasion of the homologous chromosome to form a displacement loop (D-loop), enabling the search for sequence homology and strand exchange. This critical step gives rise to various recombination intermediates that are processed into either crossover or non-crossover (NCO) products. In most organisms, the number of DSBs is greater than the number of COs and remaining DSBs are repaired by CO-independent mechanisms [17–20].

Crossover designation and resolution are mediated by a set of pro-crossover factors, including the MutSγ complex (MSH4–MSH5) which ensure the formation of stable COs [21, 22]. These events are supported by structural components of the synaptonemal complex, such as SYCP1–3 in mammals or SYP proteins in *C. elegans*, which promote chromosome pairing and synapsis [23, 24]. Together, these coordinated molecular events ensure that each homologous chromosome pair undergoes at least one CO, which is vital for proper disjunction at meiosis I and the generation of genetically diverse gametes [25].

In wild-type *C. elegans*, only one CO event is observed per pair of homologous chromosomes. This precise control of meiotic DSB repair is crucial for faithful chromosome segregation [25]. Recent studies have highlighted components of DNA damage response pathways, such as those involved in FA pathway, implicated in maintaining genome integrity during meiosis and development [26]. In this context, the FA pathway emerges as a key modulator of DSB repair fidelity, ensuring that meiotic DSBs are preferentially channeled toward HR rather than the error-prone NHEJ pathway [26].

The FA pathway is evolutionarily conserved in eukaryotes, allowing dissection and analyses using animal models such as *C. elegans*. In this model, DSB repair can be analyzed thanks to the well-defined spatio-temporal organization of the germ cells. Moreover, it is highly amenable to in vivo functional studies, including transient gene silencing by RNA interference (RNAi) and pharmacological treatments, enabling investigation of diverse biological processes such as development and apoptosis. Importantly, *C. elegans* possesses orthologs of key FA pathway genes, including *rad-51* (ortholog of human RAD51/FANCR) and *fcd-2* (ortholog of FANCD2), allowing mechanistic dissection of conserved DNA repair pathways [20, 27].

**Fig. 1:**
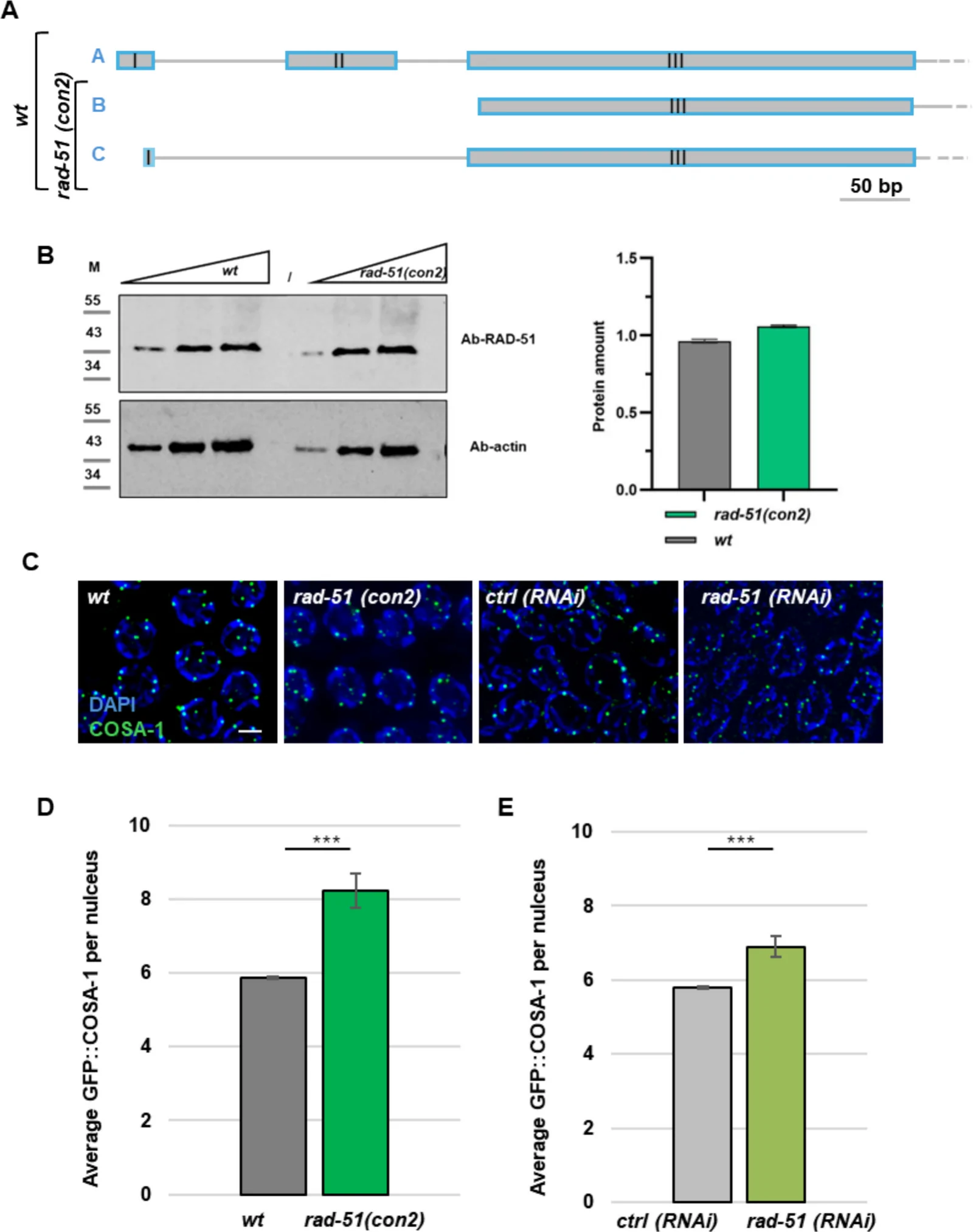
Loss of RAD-51 isoform A increases the number of COSA-1 foci. **A** Schematic representation of the N-terminal region of the three predicted wild-type *C. elegans rad-51* isoforms (A, B, and C), generated by alternative splicing. Boxes indicate the first three exons, while straight lines represent introns. The *rad-51(con2)* strain, originally designated as *rad-51(A −)* [28], was generated through CRISPR/Cas9-mediated replacement of the sequence encoding the first ATG and part of exon 1 with an engineered sequence, thereby specifically preventing translation of isoform A [28].** B** Western blot analysis showing protein abundance in *wt* and *rad-51(con2)* mutant using an anti-RAD-51 antibody. Quantification of band intensity was performed by normalization to actin. Bars indicate the mean normalized values. The Western blot shown is representative of two independent biological replicates, conducted using protein extracts derived from independent populations of the indicated strains. The second biological replicate is provided in Fig. S1. **C** Representative images of GFP::COSA-1 localization in the late pachytene regions of germ lines from worms of the indicated genotypes co-stained with anti-GFP antibody (green) and DAPI (blue). Bar, 2 μm. **D** Histograms represent the average of GFP::COSA-1 foci in late pachytene nuclei in wild-type, *rad-51(con2)* worms. **E** Histograms represent the average of GFP::COSA-1 foci in late pachytene nuclei in control (ctrl) (RNAi) and *rad-51 (RNAi)* worms. Error bars correspond to SEM calculated from at least three worms. ***p value (*t*-test) < 0.0001. Number of late pachytene nuclei scored for COSA-1 foci: *gfp::cosa-1* (*wild-type)*: 103, *rad-51(con2);gfp::cosa-1*: 93; ctrl (RNAi): 44; *rad-51* (RNAi): 59

RAD51 protein catalyzes the single-strand invasion of DNA, whereby a broken DNA molecule engages an intact duplex, promoting tightly controlled repair by HR [14]. The *C. elegans rad-51* locus is transcribed into three mRNAs that encode three isoforms, which differentiate at their N-terminal end [20, 28] (Fig. 1A).

FANCD2 is a central regulator of the FA pathway, coordinating the processing of DSBs and ICL lesions, and facilitating HR repair pathway by suppression of NHEJ [26]. Indeed, FANCD2-deficient cells show an approximately twofold reduction in HR repair efficiency of a site specific DSB [9] and an inappropriate use of NHEJ [26]. Studies in *C. elegans* and other systems have shown that the phenotypic defects associated with mutations in FANCD2 can be rescued by suppressing NHEJ, suggesting a role for FANCD2 in switching different DNA repair pathways [17–19, 26].

We previously reported that immunodetected RAD-51 and FCD-2 proteins exhibit distinct kinetics localization and do not co-localize within *C. elegans* germline cells [29]. Specifically, FCD-2 accumulation occurs only after the majority of RAD-51 foci have been dissipated, suggesting that these two factors act at different stages of DSB processing and/or are recruited to distinct repair intermediates. In line with these observations, the number of RAD-51 foci per nucleus significantly increased in the absence of FCD-2 [26], indicating that DSBs are preferentially channeled into a RAD-51-dependent pathway or alternatively a defect in RAD-51 displacement.

In this study, we provide evidence that the loss of RAD-51 isoform A enhances HR and promote the rescue of the phenotypic defects associated with the *fcd-2* null mutation in *C. elegans*. These findings reveal a previously unconsidered functional interplay between RAD51 and the Fanconi anemia pathway and suggest that targeted modulation of homologous recombination components could represent a novel therapeutic avenue to mitigate the pathological consequences of analogous mutations in Fanconi anemia patients.

## Results

### *rad-51 (con2)* mutant shows an increase in recombination frequency

We previously demonstrated that *rad-51(con2)* mutant worms, originally designated as *rad-51 (A-)* [28], in which the long *rad-51* transcript is absent (Fig. 1A), do not exhibit detectable defects in DNA repair during meiosis. This indicates that the expression of alternative RAD-51 isoforms is sufficient to sustain normal meiotic recombination processes in *C. elegans* [28].

To determine whether the absence of the long transcript affects total protein abundance, we assessed RAD-51 levels by Western blot analysis. As shown in Fig. 1B, the total RAD-51 protein levels in the *rad-51(con2)* strain are not statistically different from those in wild-type animals, indicating that the absence of the long transcript does not result in an overall reduction in protein levels. This finding is consistent with the observation that the *rad-51(con2)* mutant exhibits a frequency and distribution of RAD-51 foci similar to those of the wt, as determined by immunolocalization [28].

Considering that RAD-51 is essential for proper HR, we evaluated the HR efficiency in the *rad-51(con2)* mutant by measuring the recombination frequency within two distinct chromosomal intervals. First, *dpy-5 unc-29* on recombination chromosome I, spans a region with low recombination activity (genetic distance ~ 3.3 centimorgan (cM)), while the second, *unc-60 dpy-11* on chromosome V, corresponds to a region with higher recombination frequency (genetic distance ~ 18.8 cM). Surprisingly, the frequency of recombination in *rad-51(con2)* mutants was markedly increased across both tested chromosomal intervals, reaching 23.1% and 42%, respectively, compared to 5.2% and 18.9% observed in wild-type worms (Table 1). This reproducible increase across distinct chromosomal regions suggests an enhancement of homologous recombination efficiency in *rad-51(con2)* worms. However, we cannot exclude that fraction of the increased recombination frequencies may arise from altered of crossover interference. To further investigate the observed increase homologous recombination efficiency in recombination, we exploited the GFP-labeled COSA-1 as cytological marker to analyze the crossover-designated sites in pachytene nuclei [30]. For this purpose, we generated a *rad-51(con2);gfp::cosa-1* strain and analyzed the loading of GFP foci in the late pachytene regions of germ lines. In accordance with the collected data, *rad-51(con2)* mutant shows an increase in the COSA-1 foci per nucleus compared to wild-type worms (mean 8.2 and 5.9, respectively) (Fig. 1C–D). Notably, a similar phenotype was observed after *rad-51* silencing by RNAi in wild-type animals (Fig. 1C–E), supporting the involvement of *rad-51* isoform A in the observed phenotype. These RNAi results independently corroborate the phenotype observed in the *rad-51(con2)* mutants, underscoring the critical involvement of RAD-51 isoform A in limiting excessive crossover formation during meiotic recombination. (Fig. 1 C–E). Our findings reveal a previously unrecognized role for RAD-51 as a critical modulator of homologous recombination outcomes, challenging the established view of its function and suggesting that fine-tuning of RAD-51 isoform profile may influence the balance between crossover and alternative repair pathways.

**Table 1:**
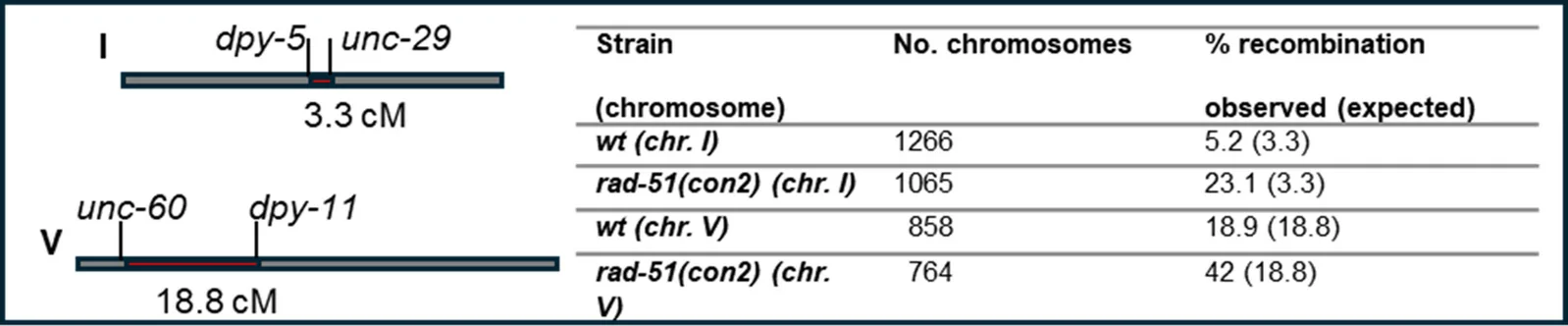
Recombination frequencies for the indicated genotypes

### Downregulation of FCD-2 recruitment in the germline and expression levels in *rad-51(con2)* mutant

In a previous study [29], we demonstrated that in wild-type *C. elegans*, FCD-2 foci accumulate only after RAD-51 foci have largely dissipated, suggesting that RAD-51 and FCD-2 act at distinct stages of DSB repair and are likely associated with different recombination intermediates [29]. To assess whether the loss of *rad-51* isoform A expression modulates the FA pathway during DSB repair, we specifically analyzed the spatiotemporal distribution of FCD-2 in the germline of *rad-51(con2)* animals. For this purpose, we generated a *flag*::*fcd-2;rad-51(con2)* strain and analyzed FCD-2 localization on DNA of germ cells.

**Fig. 2:**
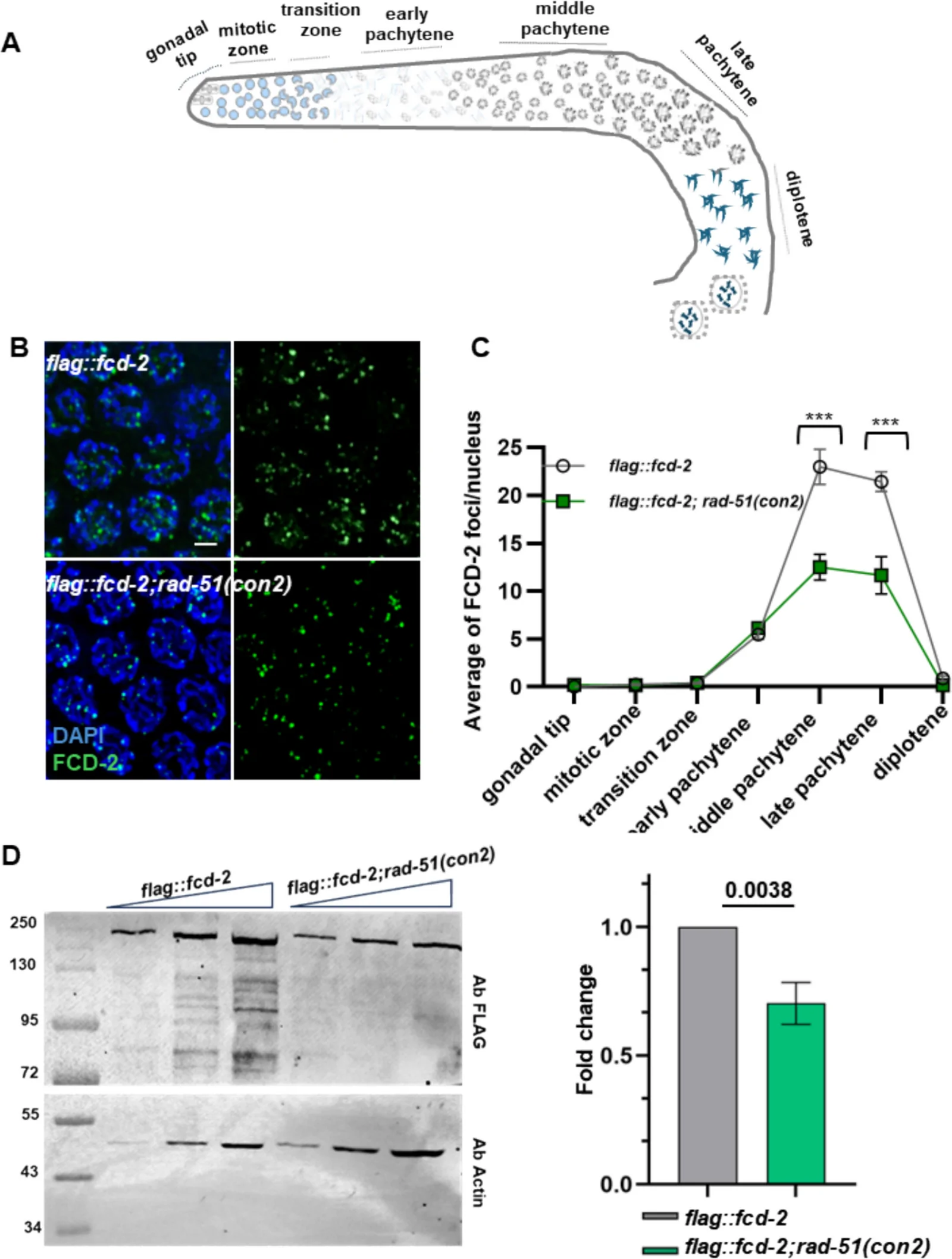
Reduction in the expression and localization of FCD-2 *in the rad-51 (con2) mutant.* **A** Schematic representation illustrating the germline zoning used to quantify *FLAG::FCD-2* foci. **B** Representative images of FLAG::FCD-2 localization in the middle pachytene regions of germ lines from worms of the indicated genotypes stained with anti-FLAG antibody (green) and DAPI (blue). Bar, 2 μm. **C** Quantification of FLAG::FCD-2 foci in the *flag::fcd-2* and *flag::fcd-2;rad-51(con2)* strains. The *y*-axis represents the average of FLAG::FCD-2 foci per nucleus. The *x*-axis represents the position (zone) along the germline. An average of 110 nuclei for each gonad region were scored for each genotype. Error bars correspond to SEM calculated from at least three independent experiments. ***p value (*t*-test) < 0.0001. **D** Western blot analysis and quantification of FLAG::FCD-2 protein levels in adult worm extracts from *flag::fcd-2 (WT) and flag::fcd-2; rad-51(con2)* animals. Different regions of the same blot were probed with the indicated antibodies. Actin was used as a loading control. Densitometric values were normalized to actin and expressed as fold change relative to WT (set to 1). Data represent mean ± SD from *n* = 3 biological replicates. Statistical significance was assessed using a two-tailed Student’s *t*-test

Analysis of FCD-2 foci revealed that the overall spatial distribution in *rad-51(con2)* germline was comparable to that observed in wild-type animals (Fig. 2A–C and S2). Strikingly, a significant reduction in the number of FCD-2 foci per nucleus was observed specifically during the middle and late pachytene stages (Fig. 2C and S2), suggesting that the loss of *rad-51* isoform A modulates the stability or recruitment of FCD-2 at sites of DNA repair during meiotic progression.

To determine whether the reduction inFCD-2 foci is due to altered protein expression levels, we analyzed FCD-2 abundance in this mutant background. Western blot analysis of total protein extracts from adult worms revealed a 30% reduction in FCD-2 protein levels in the *flag::fcd-2; rad-51(con2)* strain compared to the *flag::fcd-2* control (Fig. 2D). These findings suggest that FCD-2 downregulation may represent a compensatory or regulatory response to enhanced HR activity, potentially contributing to the modulation of DSB repair pathway choice under conditions of altered RAD-51 function.

### Loss of *rad-51* isoform A suppresses the developmental defects of *fcd-2* mutant

**Fig. 3:**
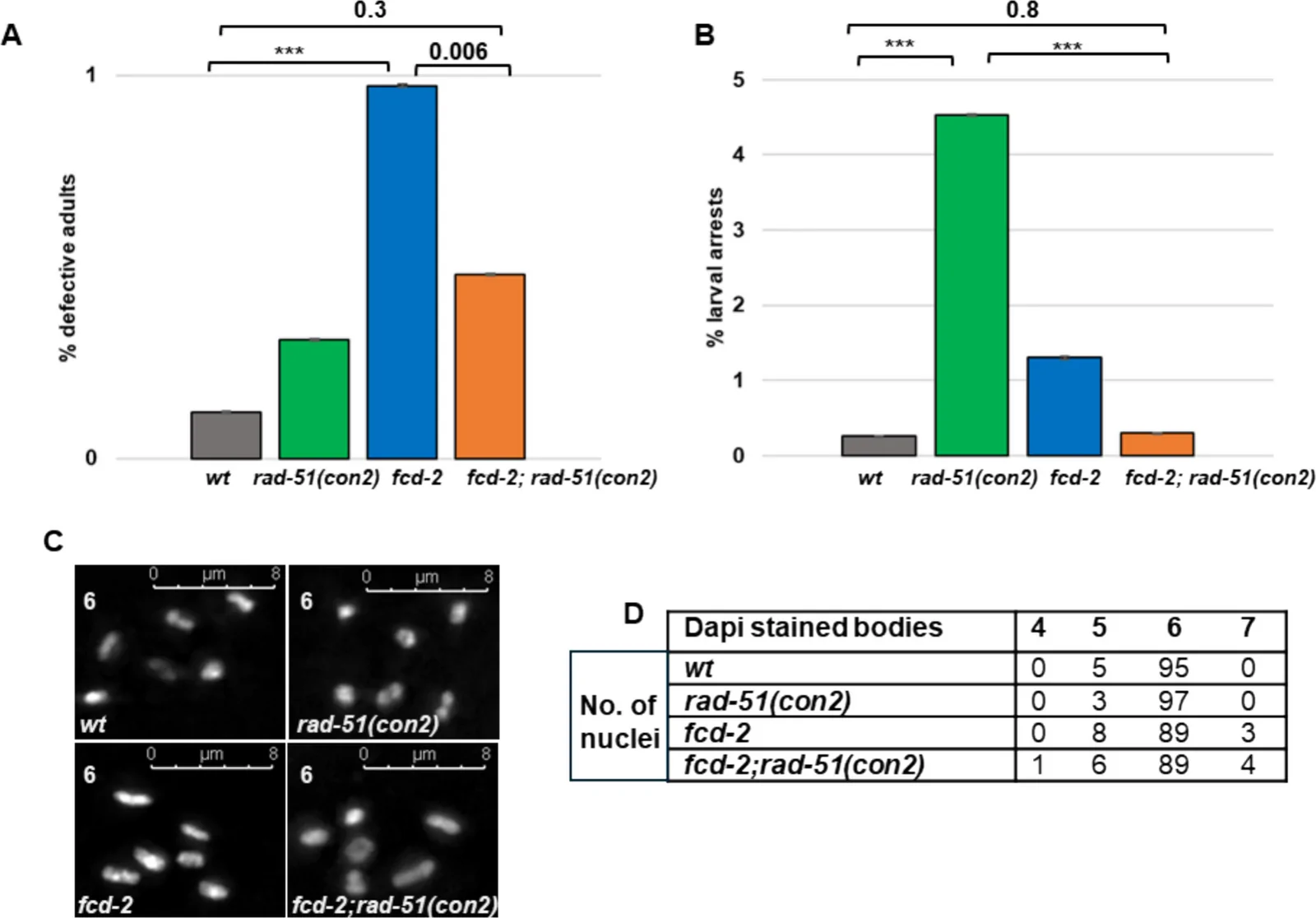
*rad-51(con2)* mutation rescues *fcd-2* mutant phenotypes. **A** Histograms show the mean percentage of defective adults for each indicated genotype, calculated by scoring developmental defects in the progeny produced by individual parental worms. **B** Histograms show the mean percentage of larval arrests for each indicated genotype, calculated by scoring larval arrests in the progeny produced by individual parental. **A**–**B** Error bars correspond to SD calculated from progeny of ten worms per each genotype. **A**–**B** ***p value (*t*-test) < 0.0001. **C** Representative images of diakinesis nuclei of the indicated genotype stained with DAPI. The number of DAPI-stained bodies is indicated at the top left of each panel. Bar, 8 μm. **D** Quantification of DAPI stained body in diakinesis nuclei in the indicated genotypes. *P* value (*t*-test) = fcd-2;*rad-51(con2) vs wild type*: 0.15

These observations prompted us to determine whether enhanced HR activity in *rad-51(con2)* might compensate for the developmental defects observed in *fcd-2* mutants, where error-prone NHEJ pathway is not properly suppressed [26]. To assess this hypothesis, we generated a *fcd-2;rad-51(con2)* double mutant. The *fcd-2;rad-51(con2)* double mutant is viable and fertile. Furthermore, developmental defects observed in *fcd-2* mutants were recovered in *fcd-2;rad-51(con2)* progeny (Table 2 and Fig. 3A). Additionally, the *fcd-2;rad-51(con2)* double mutant exhibits rescue of larval arrests compared to the *rad-51(con2)* single mutant (Table 2 and Fig. 3B), which suggests an effective DNA repair mechanism in these animals. Consistent with a correct meiotic chromosome segregation, six DAPI-stained bodies, corresponding to the expected six bivalents, were observed in most diakinesis nuclei from *fcd-2;rad-51(con2)* germlines (Fig. 3C–D), indicating the correct formation and pairing of the homologous chromosomes in the *fcd-2;rad-51(con2)* strain**.** Taken together, these results demonstrate that the loss of *rad-51* long isoform A promotes the rescue of developmental aberration in a *fcd-2*-depleted background.

**Table 2:** Screening of *wild-type*,* rad-51(con2)*,* fcd-2*, and *fcd-2;rad-51(con2)* phenotype

*Genotype*	*wt*	*rad-51(con2)*	*fcd-2*	*fcd-2;rad-51(con2)*
Parental worms	10	10	10	10
Laid eggs	2728	2321	2002	2504
Dead embryos (%)	22 (0.81)	19 (0.82)	29(1.45)	29 (1.16)
Hatched eggs	2706	2303	1973	2475
Larval arrests (%)	7 (0.26)	104 (4.52)	23 (1.16)	7 (0.28)
Defective adults (%)	3 (0.1)	7 (0.32)	19 (0.97)	12 (0.49)

### Loss of *rad-51* isoform A restores the correct use of NHEJ in *fcd-2* mutant

Wild-type *C. elegans* diakinesis oocytes present six DAPI-stained bodies or bivalents (representing the six homologs held together by chiasmata). In contrast, oocytes from *syp-2* mutant, defective in the formation of the synaptonemal complex [24], or from *msh-4*/*MSH4* mutants, in which CO is impaired [21], generally contain 12 unpaired chromosomes (univalents). In a previous study [26], we showed that under conditions where homologous chromosomes are unavailable for recombination, *fcd-2* mutant oocytes display between 6 and 12 DAPI-stained bodies (*syp-2;fcd-2* and *msh-4;fcd-2* double mutants). This reflects the non-covalent aggregation of non-homologous chromosomes, indicating a failure in proper homologous recombination [26]. Moreover, deletion of *lig-4* (essential for NHEJ) in the *syp-2;fcd-2* and *msh-4;fcd-2* backgrounds results in a shift toward 12 or more DAPI-stained bodies at diakinesis, indicating that the non-covalent aggregation results from enhanced NHEJ-mediated repair activity [26]. The presence of more than 12 DAPI-stained bodies at diakinesis indicates inefficient intersister HR repair in the *fcd-2* mutant [26].

**Fig. 4:**
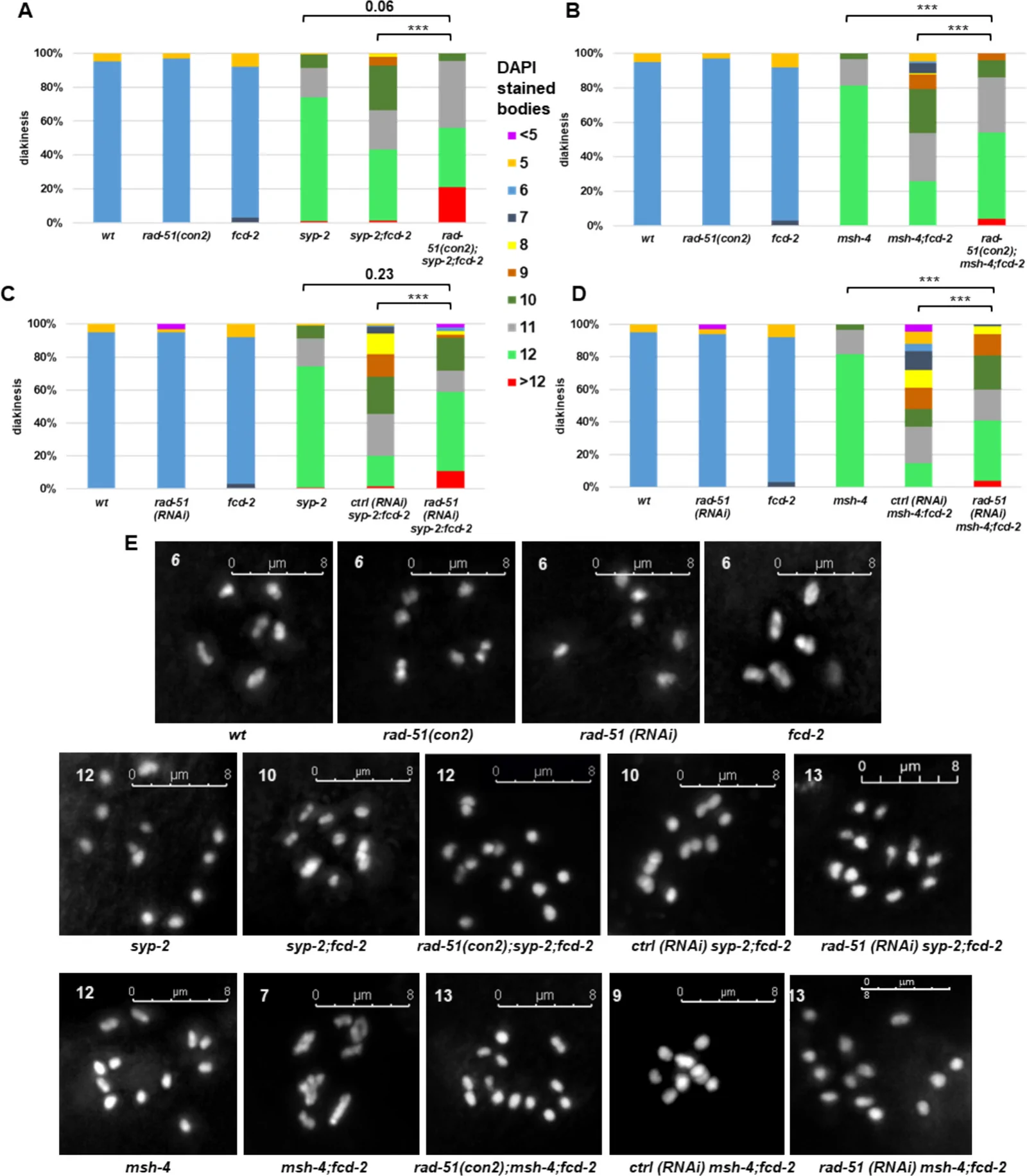
Loss of RAD-51 isoform A suppresses the promiscuous use of NHEJ in the *fcd-2* mutant. **A**–**D** Quantification of DAPI-stained bodies. Number of observed diakinesis nuclei: *wt* = 100; *rad-51(con2)* = 100; *rad-51 (RNAi)* = 100; *fcd-2* = 100; *syp-2* = 116*, msh-4* = 92*; syp-2;fcd-2* = 137, *msh-4;fcd-2* = *109*,* rad-51(con2);syp-2;fcd-2* = 99, *rad-51(con2);msh-4;fcd-2* = *100*,* ctrl (RNAi) syp-2;fcd-2* = 125,* ctrl (RNAi) msh-4;fcd-2* = 110,* rad-51 (RNAi) syp-2;fcd-2* = *46*,* rad-51 (RNAi) msh-4;fcd-2* = 100. **E** Representative images of diakinesis nuclei of the indicated genotype stained with DAPI. The number of DAPI-stained bodies is indicated at the top left of each panel. Bar, 8 μm. ***p value *t*-test)

To investigate whether the loss of *rad-51* long isoform could suppress chromosomes aggregation in absence of homologous chromosomes observed in *fcd-2* animals, we generated *rad-51(con2);syp-2;fcd-2* and *rad-51(con2);msh-4;fcd-2* triple mutants. As shown in Fig. 4, the number of DAPI-stained bodies is predominantly 12 in both *rad-51(con2);syp-2;fcd-2* and *rad-51(con2);msh-4;fcd-2* oocytes (Fig. 4A, B, E), indicating strong suppression of non-covalent aggregation of non-homologous chromosomes, and suggesting that NHEJ is not aberrantly activated in these triple mutants. Similar results were observed after silencing of the *rad-51* gene by RNA*i* in *syp-2;fcd-2* and *msh-4;fcd-2* worms (Fig. 4C, D, E), supporting the idea that RAD-51 levels play a critical role in regulating the balance between alternative DNA repair pathways in absence of *FCD-2*.

## Discussion

RAD-51 and FCD-2 are key factors involved in the repair of DSBs, and their dysfunction in humans is associated with FA, a disorder characterized by genome instability and developmental defects. Both proteins are typically implicated in directing repair toward high-fidelity HR pathway [17–19, 26]. However, the lack of temporal and spatial colocalization of RAD-51 and FCD-2 in the *C. elegans* germline [29] suggests that these proteins may function at distinct stages of the repair process or act on different DNA substrates.

In this study, we show an increase in recombination frequency in *rad-51(con2)* strain, without impairing meiotic progression or fertility. The recombination frequency has been evaluated on two different chromosomes and in two genomic intervals characterized by distinct recombination activities. It is also supported by an independent cytological assay data. These results suggest an enhancement of recombination efficiency in absence of RAD-51 isoform A. In line with this, it remains possible that the loss of isoform A disrupts crossover interference. This data challenges the prevailing view that higher RAD-51 levels directly correlate with increased HR efficiency. Instead, our data support a model in which the RAD-51 isoform profile is crucial for orchestrating the balance between alternative DNA repair pathways. Specifically, this model suggests that the loss of RAD-51 isoform A shifts the balance between repair pathways, likely by reducing competition for recombination intermediates and enabling pro-crossover factors to stabilize them. Furthermore, we demonstrate that the loss of *rad-51* A isoform mitigates the DNA repair defects observed in *fcd-2* mutant. Notably, the *fcd-2;rad-51(con2)* strain exhibits reduced larval arrests and partial rescue of aberrant adult morphological phenotypes. This genetic interaction also suppresses the aberrant chromosomal fusion typically observed in *fcd-2* mutant, suggesting a restored equilibrium between HR and NHEJ. In addition, in the *rad-51(con2)* background we observed a reduction of FCD-2 on nuclei, while total RAD-51 protein levels remained constant. This suggests a feedback loop between HR dynamics and FA pathway regulation.

## Conclusions

In conclusion, our findings underscore the pivotal role of RAD-51 in regulating the delicate balance between HR and NHEJ, and support the idea that modulating DNA repair pathway may represent a promising strategy for alleviating FA-associated genomic instability. Future studies will be essential to dissected this regulatory network may provide broader insights into how cells coordinate DNA repair processes to maintain genomic integrity. This could inform therapeutic strategies for FA and other disorders associated with defective DSB repair.

## Methods

### Strains

Nematodes (listed in Table S1) were cultured at 20 °C on NGM plates with *Escherichia coli* OP50 as food source, according to standard methods [31]. The PCRs to identify mutations and the double/triple mutants were performed with the primers listed in Table S2.

### Immunostaining in *C. elegans* germline

Gonads of 20 h post L4 larval stage worms were dissected in M9 buffer (0.6% Na2HPO4, 0.5% NaCl, 0.3% H2PO4 and 1 mM MgSO4) on Poly-Lysine glass slides. The specimens were freeze-cracked in liquid nitrogen, sequentially immersed, at − 20 °C, in methanol, methanol/acetone (1:1) and acetone for 5 min each, and washed three times in PBS, for 5 min. Slides were blocked in 0.3% BSA in PBS for 30 min at 37 °C in a humid chamber. The primary antibodies used in this study were goat anti-GFP and mouse anti-FLAG diluted 1:150 and 1: 200, respectively, in Ab buffer (1% BSA, 0.1% Tween-20, 0.05% sodium azide in PBS). Slides were incubated for 90 min at room temperature followed by three washes in PBS, 5 min each. Secondary antibodies were conjugated with donkey anti-goat Alexa Fluor 488 and donkey anti-mouse Alexa Fluor 488 1:400 dilution. Slides were incubated for 60 min in the dark at room temperature, followed by three washes in PBS + 0.1% Tween-20 for 5 min each time. Slides were mounted with Prolong Gold Antifade reagent with DAPI (Life Technologies) [32]. Foci/nucleus were quantified manually from deconvolved three-dimensional (3D) stacks. Images shown are projections through 3D data stacks.

### Proteins extraction from adult worms

Adult worms were collected by washing NGM plates with ddH2O in a centrifuge tube and pelleted by centrifugation at 400 × g for 2 min at 4 °C. Drops of the adult worm pellet were placed in a cool mortar containing liquid nitrogen to obtain worm beads. These beads were thoroughly ground with a pre-cooled pestle until a fine powder was obtained. A total of 0.150 g of frozen worm powder was resuspended in 400 µL of lysis buffer (250 mM Tris pH 7.5 + 1X protein inhibitor (Roche). The mixture was vortexed briefly to ensure complete homogenization, while maintaining cold conditions throughout the procedure. Protein concentration in the resulting lysates was determined using the Bio-Rad Protein Assay reagent, according to the manufacturer’s instructions.

### Western blot

Different amounts of protein extracts were analyzed by western blot. Samples were run on 8% polyacrylamide gel in 1 × Tris–glycine-SDS Running buffer (25 mM Tris, 192 mM glycine and 0.1% SDS, pH 8.3). After electrophoresis, proteins were transferred onto PVDF filters (Bio-Rad, Hercules, CA, USA) using the Trans-Blot1 TurboTM Blotting System (Bio-Rad). Subsequently, the PVDF membrane was incubated with the PVDF blocking reagent (PBST supplemented with 5% milk; Bio-Rad) for 60 min at room temperature (RT) and washed thrice in PBST for 5 min with shaking. After blocking the PVDF membrane was cut in two parts and incubated of 1 h at RT with the appropriate primary antibodies diluted in PBST: rabbit anti-RAD-51 antibody (1:1000), mouse anti-FLAG antibody (Abcam; 1:10,000), or anti-actin antibody (Abcam; 1:3000). The membranes were subsequently incubated for 1 h at RT with an anti-rabbit secondary (Pierce 1:50,000) or anti-mouse secondary antibody (Bio-Rad; 1:3000). The protein analysis was performed using ECL-PLUS kit (GE-Healthcare) according to the manufacturer’s instructions, and signals were acquired using an iBright imaging system (Thermo fisher Scientific) [33]. Densitometric data were acquired using the iBrigh Analysis Software. Normalized densitometric values were expressed as fold change relative to the wild type (set to 1) and all data processing, graphing and statistical analyses were performed using GraphPad Prism [33].

### Phenotype screening

Young adult hermaphrodite worms were individually transferred to fresh NGM plates and incubated at 20 °C to lay eggs for 3 days. Worms were transferred every 12 h onto fresh plates until all fertilized eggs were laid. Each plate was monitored for 24/72 h to analyze the following parameters among the progeny: embryonic lethality, larval arrests and developmental defects. The percentage of embryonic lethality is calculated as the ratio of unviable eggs on laid eggs. The percentage of larval arrests or developmental defects is calculated as the ratio of larval arrests/developmental defects on the hatched eggs [28].

### RNA interference

RNA*i* was performed by feeding worms at L4 stage with HT115 bacteria transformed with the control plasmid L4440 or bacteria expressing dsRNA corresponding to the *rad-51* coding region worms [34, 35] using clones from the Ahringer library (Gurdon Institute, Cambridge, UK) [36]. DAPI stained body in diakinesis nuclei and the COSA-1 levels were analyzed after 6 days in the progeny.

### Analysis of DAPI-stained bodies in the germline nuclei

About 10 adult nematodes were placed in a drop of M9 buffer on glass slides, permeabilized and fixed with 8 mL of 100% EtOH and directly mounted in 8 mL DAPI (2 ng/ml) diluted in M9 buffer [37]. Numbers of scored nuclei are indicated in the legend of charts.

### Image collection and processing

Fluorescence imaging was performed using a Leica DM6 microscope equipped with a Hamamatsu digital camera, operated via Leica LAS X 6000 software. Image acquisition was processed and deconvoluted using Leica LAS X software (version 3.0.0.15697). Quantitative analyses of COSA-1 foci, FCD-2 foci and DAPI-stained chromatin along the gonadal axis were conducted on Z-stack images. Optical sections were captured at intervals of 0.18 µm for COSA-1 and FCD-2 foci and 0.50 µm for DAPI-stained bodies. The images presented represent maximum-intensity projections of the Z-stacks.

### Statistical tools

Statistical analyses for independent samples were computed through Student’s *t*-test. Student’s *t*-test for independent samples was used for the analysis of COSA-1 foci, FCD-2 foci, larval arrests, and developmental defects and diakinesis.

## Supplementary Information

Supplementary Material 1. Table S1. List of all strains used in this study. Table S2. List of all primers used in this study. Fig S1. Western blot analysis showing RAD-51 protein abundance in wt and rad-51mutants using an anti-RAD-51 antibody. Band intensity was quantified by densitometric analysis and normalized to actin. Bars indicate the mean normalized values. Fig S2. Localization of FCD-2 along the germline in rad-51mutant. Representative projection images illustrating the germline zoning used to quantify FLAG::FCD-2 foci in dissected gonad from flag::fcd-2 and flag::fcd-2;rad-51hermaphrodite worms, stained with anti-FLAG antibodyand DAPI. Scale bar = 20 µm.

## Data Availability

No datasets were generated or analysed during the current study.

## Abbreviations

BSA: Bovine serum albumin
cM: Centimorgan
CO: Crossover
DAPI: 4′,6-Diamidino-2-phenylindole
D-loop: Displacement loop
DSB: Double strand break
FA: Fanconi anemia
GFP: Green fluorescent protein
HR: Homologous recombination
ICL: Inter-strand cross-link
NCO: Non-crossover
NHEJ: Non-homologous end joining
NGM: Nematode growth medium
PCR: Polymerase chain reaction
PBS: Phosphate-buffered saline
PBST: Phosphate-buffered saline containing 0.1% Tween® 20
qPCR/RT: Quantitative PCR
RNAi: RNA interference
RT: Room temperature
SDS: Sodio dodecyl sulphate
S phase: Synthesis phase
