# Supplementary material for: Loss of the RAD-51 isoform A redirects DNA repair and preserves genome stability in FANCD2-deficient Caenorhabditis elegans
Source: BMC Biol. 2026 Jun 23;24:196. doi: 10.1186/s12915-026-02652-1 (PMC13587426; doi:10.1186/s12915-026-02652-1)
Supplement: Supplementary file 1 [file 12915_2026_2652_MOESM1_ESM.docx]

***Supplementary Information***

***Loss of the RAD-51 isoform A* redirects DNA repair and preserves genome stability in FANCD2-deficient** ***Caenorhabditis elegans*** (*Alessandra Masullo et al.)*

| **Strain** | **genotype** | **Source** |
| --- | --- | --- |
| N2 | Wild type, Bristol | CGC |
| NB105 | *fcd-2(tm1298) IV* | CGC |
| DR102 | *dpy-5(e61) unc-29(e403) I* | CGC |
| BC3217 | *unc-60(m35) dpy-11(e224) V* | CGC |
| AV630 | *mels8 [pie-1p::gfp::cosa-1 + unc-119 (+)] II* | CGC |
| AV276 | *syp-2(ok307) V/nT1 [unc-?(n754) let-? qls 50] (IV;V)* | M. Colaiacovo |
| AV308 | *him-14(it21)/mnCI [dpy-10(e128) unc-52 (e444) II* | CGC |
| AAD1 | *fcd-2(tm1298) IV; syp-2(ok307)V /nT1 [unc-?(n754) let-? qls 50] (IV;V)* | A. Adamo |
| AAD2 | *fcd-2(tm1298) IV; him-14(it21)/ mnCI [dpy-10(e128) unc-52 (e444) II* | A. Adamo |
| AAD3 | *fcd-2(con1)[3xflag::fcd-2] IV* | A. Adamo |
| AAD4 | *rad-51(con2) IV* (previously designated as *rad-51 (A-)* IV [28]) | A. Adamo |
| AAD5 | *rad-51(con2) IV; dpy-5(e61) unc-29(e403) I* | This study |
| AAD6 | *rad-51(con2) IV; unc-60(m35) dpy-11(e224)V* | This study |
| AAD7 | *rad-51(con2) IV; mels8 [pie-1p::gfp::cosa-1 + unc-119 (+)] II* | This study |
| AAD8 | *fcd-2(con1)[3xflag::fcd-2] rad-51(con2) IV* | This study |
| AAD9 | *fcd-2(tm1298) rad-51(con2) IV* | This study |
| AAD10 | *rad-51(con2) fcd-2 (tm1298) (IV);syp-2(ok307)V /nT1 [unc-?(n754) let-? qls 50] (IV;V)* | This study |
| AAD11 | *rad-51(con2) fcd-2 (tm1298) (IV); him-14(it21)/ mnCI [dpy-10(e128) unc-52 (e444) II* | This study |

**Table S1. List of all strains used in this study**

| **PRIMER** | **GENE** | **SEQUENCE (5’- 3’)** |
| --- | --- | --- |
| Upper-rad-51 | *rad-51 (F)* | TATGGGACAATCTTGGGG |
| Upper-rad-51(con2) | *rad-51[in rad-51(con2) strain] (F)* | ACGAAGTTATGGCTGCTCG |
| Lower- rad-51 | *rad-51(R)* | ACGAAGTTATGGCTGCTCG |
| Upper-fcd-2 | *fcd-2* | TCGCTCCGCCCTCTTTTCTA |
| Lower-fcd-2 | *fcd-2* | CGACGAGCAGCTAACAACATTGG |
| Upper-GFP | *gfp [in AV630 strain] (F)* | GCCCGAAGGTTATGTACAGG |
| Lower-GFP | *gfp [in AV630 strain] (R)* | CCCGGGCATTTGTATAGTTC |
| Upper-3xFLAG- fcd-2 | *fcd-2* | TTCCCGCCATGGAGCATG |
| Lower-3xFLAG-fcd-2 | *3xflag[in 3xflag::fcd-2]* | GTGGCCATCATCAGGATC |

**Table S2. List of all primers used in this study**

**Fig S1.** Western blot analysis showing RAD-51 protein abundance in *wt* and *rad-51(con2)* mutants using an anti-RAD-51 antibody. Band intensity was quantified by densitometric analysis and normalized to actin. Bars indicate the mean normalized values.

**Fig S2. Localization of FCD-2 along the germline in *rad-51(con2)* mutant**

Representative projection images illustrating the germline zoning used to quantify FLAG::FCD-2 foci in dissected gonad from *flag::fcd-2* and *flag::fcd-2;rad-51(con2)* hermaphrodite worms, stained with anti-FLAG antibody (green) and DAPI (blue). Scale bar = 20 µm**.**
